# Mid-to-late Holocene climate variability in coastal East Asia and its impact on ancient Korean societies

**DOI:** 10.1038/s41598-023-42551-x

**Published:** 2023-09-16

**Authors:** Jungjae Park, Junbeom Bahk, Jinheum Park, Hyejin Kim, Jieun Choi

**Affiliations:** 1 Department of Geography, Seoul National University, 1 Gwanak-Ro, Gwanak-Gu, Seoul, 08826 Republic of Korea; 2 Institute for Korean Regional Studies, Seoul National University, 1 Gwanak-Ro, Gwanak-Gu, Seoul, 08826 Republic of Korea; 3 Department of Geography, University of Cambridge, Downing Place, Cambridge, CB2 3EN UK

**Keywords:** Ecology, Palaeoecology, Climate sciences, Palaeoclimate

## Abstract

The sustainability of human societies is contingent upon our ability to accurately predict the effects of future climate change on the global environment and humanity. Wise responses to forthcoming environmental alterations require extensive knowledge from historical precedents. However, in coastal East Asia, a region with a long history of agriculture, it is challenging to obtain paleoenvironmental proxy data without anthropogenic disturbances that can be used to assess the impact of late Holocene climate change on local communities. This study introduces a high-resolution multi-proxy sedimentary record from an isolated crater in Jeju Island, Korea, to elucidate the mechanisms underlying mid-to-late Holocene climate change and its impacts on ancient societies. Our findings suggest that hydroclimate changes were predominantly governed by sea surface temperature fluctuations in the western tropical Pacific, with low-frequency variability in solar activity and a decrease in summer insolation identified as primary drivers of temperature change. Moreover, ancient societies on the Korean peninsula were significantly affected by recurring cooling events, including the 2.8 ka event, 2.3 ka event, Late Antique Little Ice Age, maunder minimum, and others.

## Introduction

The persistent progression of global warming and the accelerating pace of environmental change pose serious threats to human survival, leading to increased interest in understanding how past climate changes influenced ancient societies and their coping mechanisms. The coastal regions of East Asia, including eastern China, the Korean Peninsula, and Japan, have been populated for thousands of years, making them ideal for such research. By reconstructing past climates and vegetation, then correlating those data with the region’s abundant archaeological and historical records, we can acquire extensive information about the potential consequences of future climate change on humans and societies.

To explore past climate changes, scientists analyze various natural samples such as glaciers, stalagmites, tree rings, and lake sediments. However, in coastal East Asia, it is particularly challenging to find suitable samples for paleoenvironmental research. Although glaciers and stalagmites provide detailed, long-term information regarding past climate variability, their locations in remote areas (e.g., poles and karst mountains) limit their usefulness with respect to analyzing local climate variations that could have influenced ancient societies^1,2^.

Furthermore, tree rings offer precise dating, which is crucial for establishing the relationship between Holocene climate change and local social responses. However, trees that are sensitive to climate change are generally conifers that thrive in arid regions with < 800 mm of annual precipitation^3^. Paleoclimate information from trees in agriculturally favorable areas is sparse. Thus, except for a few limestone caves^4–6^, wetlands such as lakes and swamps are the only viable sites for Holocene climate investigation in coastal East Asia.

The main challenge lies in differentiating climate change signals from human impacts in sediment proxy data, particularly in regions such as East Asia where a substantial human population in the late Holocene may have influenced these records. For example, pollen data reported from a river floodplain in the southern part of the Korean Peninsula^2^ clearly showed decreases in the proportion of tree pollen at 2800 and 2300 cal year BP, but there have been differing opinions about this change. The authors saw this change as a result of abrupt short-term drying due to reduced solar activity^2^, while other researchers interpreted it as a result of human activities^7^.

Therefore, less anthropogenically impacted alpine lakes and wetlands, such as our study site (Dongsuak crater, situated in Mt. Hallasan National Park) are preferred coring targets. The Dongsuak crater, primarily covered by forest and containing wetlands, offers sediment samples that have remained largely undisturbed by human activities, a rare find in densely populated coastal regions of East Asia with extensive habitation histories. Intact paleoclimate records from this study will also provide a unique opportunity to test the hypothesis of Park et al. that late Holocene climate deterioration led to a southward migration of agriculturalists in coastal East Asia^2^.

Pollen, a popular sediment proxy data source, is limited by the ability to determine whether vegetation change resulted from temperature or precipitation variations. To address this, we reconstructed shifts in these climate factors predominantly on the basis of pollen data and charcoal accumulation rates (CARs). We sought to thoroughly explore the effects of climate change on human societies.

This paper presents a new high-resolution multi-proxy record (pollen, charcoal, total organic carbon [TOC], and magnetic susceptibility [MS]) of the Holocene using a sediment core from Dongsuak swamp on Jeju Island, South Korea. The research aims to (1) reconstruct the mid-to-late Holocene climate change history in the study area, (2) understand the mechanisms underlying hydroclimate change and temperature variations during this period, (3) examine abrupt short-term cooling and/or drying events, and (4) explore the possible effects of these events on ancient human societies.

## Study area

### Site description

**Figure 1:**
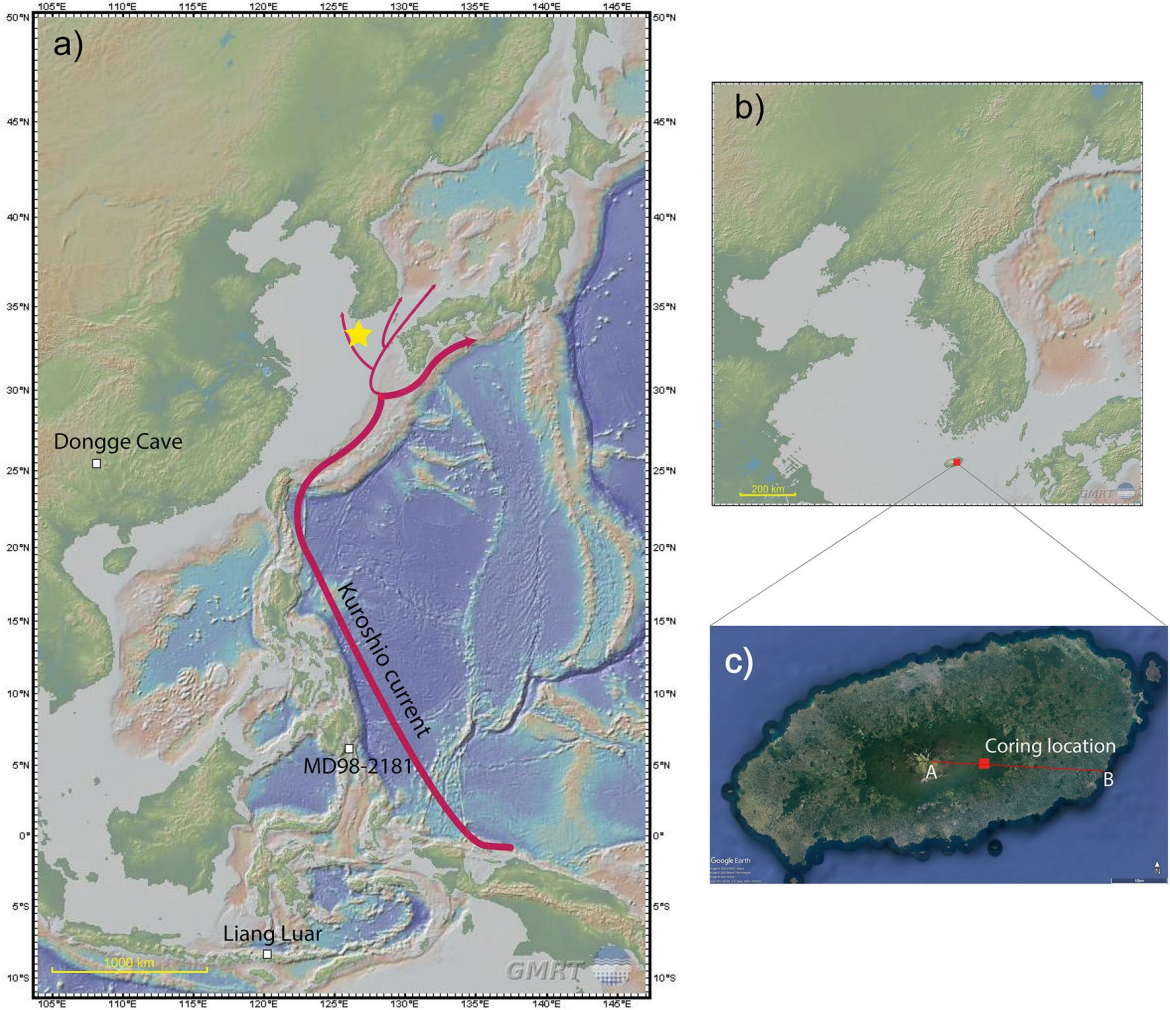
(**a**) Locations of the study site (yellow star) and the paleoclimate records used or mentioned in this study: site MD98-2181, Mindanao, Philippines^24^ (Fig. 5), Liang Luar Cave, southeastern Indonesia^31^ (Fig. 7), Dongge Cave, southern China^59^. (**b**) Coring site location (red square), Dongsuak crater, Jeju Island, South Korea. These location maps were created using the GMRT Map tool (www.gmrt.org/GMRTMapTool/) ^85^. (**c**) Coring location (red square). Line AB indicates the position of the vegetation profile shown in Fig. 2a. This image was created using Google Earth (www.google.co.kr/intl/ko/earth/).

The Dongsuak crater (33° 21′ 41ʺ N, 126° 37′40ʺ E) is located in the eastern region of Jeju Island, South Korea (Fig. 1). Jeju Island is a shield volcano that emerged from the continental shelf of the Yellow Sea; it comprises layers of basaltic lava and some pyroclastic deposits. The island contains > 450 Quaternary satellite cones, including cinder (scoria) cones, lava cones/domes, and hydromagmatic tuff rings/cones. The centerpiece of the island, Mt. Hallasan, is 1950 m high. The Dongsuak crater, positioned within a parasitic cone on the eastern flank of the primary volcano, is mostly surrounded by permeable basalt that limits surface water.

Nevertheless, impermeable fine clastic sediments, trapped within craters such as Dongsuak, prevent water from seeping into the ground. The Dongsuak crater, part of Mt. Hallasan National Park, is currently safeguarded under the Korea Cultural Heritage Protection Act. The crater is located ~ 700 m above sea level, with a height of ~ 100 m and a circumference of ~ 1800 m. Its northern slope is gradual, whereas its western slope forms a steep, horseshoe-shaped crater that extends southeastward^8^. Pollen and macrofossil evidence indicate continuous sedimentation over the past 7000 years^9^; consequently, the crater has transitioned from a lake to a swamp, and gradually to a meadow from the edges inward. It currently contains a swamp with a circumference of ~ 220 m^10^.

### Climate

Korea's climate is distinguished by four distinct seasons, marked by significant variations in monthly average temperatures between summer and winter. Most rainfall occurs during the summer months. The southeast summer monsoon introduces hot, humid conditions to the Korean peninsula, whereas the northwest winter monsoon brings cold, dry weather. During winter, a strong high-pressure cell over continental Siberia triggers clockwise air circulation that flows southeastward over the peninsula. In contrast, during summer, the wind direction reverses because inland areas experience more warming than the sea, leading to the summer monsoon-mediated transport of warm moisture from the sea to the peninsula. However, Jeju Island, our study site, experiences a mild, oceanic climate throughout the year, with a less extreme annual temperature range relative to the peninsula.

**Figure 2:**
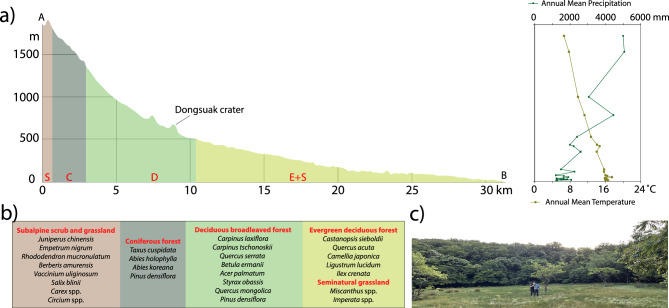
(**a**) Vegetation profile on the eastern slope of Jeju Island and altitudinal variations in annual mean temperature and precipitation^86^. (**b**) Dominant plants in each vegetation zone. (**c**) A photograph from inside the crater. It was taken by Jieun Choi.

The nearby Seongsanpo station records a mean January temperature of 5.4 °C and a mean August temperature of 26.5 °C. The region receives annual mean rainfall of 2030 mm, thus ranking second among the 74 stations in South Korea^11^. Because of orographic effects, the slopes of Mt. Hallasan experience significant variation in annual rainfall (Fig. 2a), with high precipitation primarily on the eastern and southern flanks of the island. This pattern is partially attributed to the maritime effects of the warm Tsushima Current, a subset of the larger Kuroshio Current (Fig. 1), which weakens the winter monsoon, thereby yielding relatively mild and humid conditions on the island during winter. The effects of tropical cyclones, or typhoons, are particularly visible on Jeju Island during the summer.

### Vegetation

Vegetation on Jeju Island displays significant altitude-based variations, which have been the subject of numerous studies (Fig. 2b)^12–14^. The eastern slope of Mt. Hallasan contains various forest types, including evergreen broadleaved forests (ca. 0–500 m above sea level), deciduous broadleaved forests (ca. 500–1350 m), mixed deciduous broadleaved-coniferous forests (ca. 1350–1500 m), coniferous forests (ca. 1500–1800 m), and subalpine scrub and grassland (ca. 1800–1950 m).

Several lower elevation regions have been repurposed for agriculture^12,14^. Historical records indicate that over the past millennium, semi-natural lowland grasslands have been used to raise horses and cattle. The grazed areas are predominantly populated by species such as *Trifolium repens*, *Botrychium virginianum*, *Rosa multiflora*, *Miscanthus sinensis*, and *Imperata cylindrica*^15^.

The evergreen broadleaved forest mainly consists of species such as *Castanopsis sieboldii*, *Quercus acuta*, *Distylium racemosum*, *Camellia japonica*, *Eurya japonica*, *Ligustrum lucidum*, *Ilex crenata*, and *Daphniphyllum macropodum*; the deciduous broadleaved forest is populated by *Carpinus laxiflora*, *C. tschonoskii*, *Quercus serrata*, *Betula ermanii*, *Acer palmatum*, and *Styrax obassia*^14^. The mixed deciduous broadleaved-coniferous forest is dominated by *Quercus mongolica* and *Pinus densiflora*; *Taxus cuspidata*, *Abies holophylla*, *Abies koreana*, and *Pinus densiflora* are the primary species found in the coniferous forest^16^. The scrub at the peak of Mt. Hallasan primarily consists of *Juniperus chinensis*, *Empetrum nigrum*, *Rhododendron mucronulatum*, *Berberis amurensis*, *Vaccinium uliginosum*, *Salix blinii*, and herbs such as *Carex siderosticta*, *Tofieldia fauriei*, *Cirsium japonicum*, and *Duchesnea chrysantha*^12^.

The Dongsuak swamp currently contains various plant species, including (in descending order of frequency) *Isachne globosa*, *Juncus effusus*, *Persicaria sieboldii*, *Scirpus fluviatilis*, and *Carex heterolepis*. Less commonly encountered species include *Eleocharis acicularis*, *Scirpus triangulates*, *Eriocaulon sieboldianum*, *Stellaria alsine*, and *Aneilema keisak*. However, because of human activities and the resulting clastic influx, the swamp is gradually transitioning into a meadow. The ecotone between the forest and meadow features species such as *Rosa multiflora* and *Viola verecunda*^17^. The inner slopes of the crater are dominated by deciduous broadleaved trees such as *Carpinus laxiflora*, *Carpinus tschonoskii*, *Quercus Serrata*, and *Styrax japonica*, as well as evergreen broadleaved trees such as *Neolitsea sericea*^18^.

## Results and discussion

### Chronology and stratigraphy

**Figure 3:**
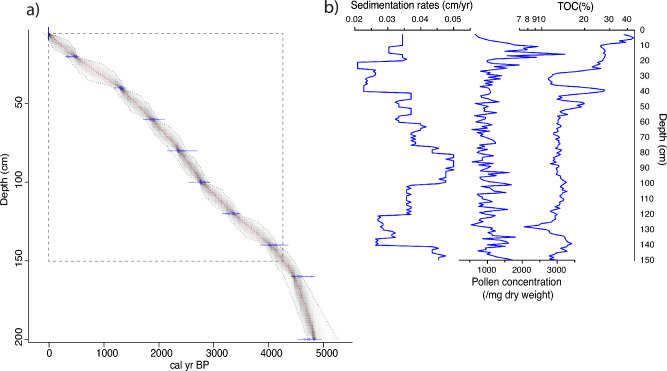
(**a**) The Dongsuak sediment core age depth profile. The optimal age depth model (red dotted line), with a 95% confidence interval (gray dotted line), was established based on Bayesian principles using rbacon 3.0.0 software. (**b**) Sedimentation rates, pollen concentration, and total organic carbon (TOC) in Dongsuak sediments. This diagram was generated using pro Fit 7.0.19 software (www.quansoft.com).

This study established a depth-age model for our 3-m-long core, founded on 10 radiocarbon dates (Table 1). The deposition rate of the Dongsuak sediments was relatively constant, suggesting that the swamp's sedimentary environments remained stable during the mid-to-late Holocene. Paleoenvironmental multi-proxy data were obtained from the upper 150 cm of the sediment core (Fig. 3). Sedimentation rates from the 150 cm depth to the surface fluctuated between 0.2 mm/year and 0.5 mm/year, averaging 0.36 mm/year. Sediments below 50 cm depth were relatively coarse, containing even sandy grains with low organic contents. However, sediments above this depth, primarily composed of fine silt materials, were characterized by decreased deposition rates, as well as increasing pollen concentrations and TOC percentages. TOC values from 150 cm depth to 55 cm depth were mostly below 15%. However, there was a substantial increase from the 27 cm depth upwards, exceeding 40% at the topmost layer.

**Table 1:** Radiocarbon dates for Dongsuak sediments. The data were calibrated using rbacon 3.0.0 software^72^ and the IntCal20 dataset^73^.

Sample depth (cm)	Material dated	Laboratory no.	δ^13^C ‰)	Age (^14^C year BP)	Two σ age range (cal year BP)	Probability (%)
5	Plant remains	BETA-597144	− 29.4	101.38 ± 0.38 pMC	− 5 to − 6	95.4
20	Bulk sediments	BETA-601089	− 28.8	400 ± 30	513–428	73.8
					376–325	21.6
40	Bulk sediments	BETA-597145	− 27.4	1400 ± 30	1350–1284	95.4
60	Bulk sediments	BETA-601090	− 23.6	1960 ± 30	1951–1820	87.5
					1991–1957	7.9
80	Bulk sediments	BETA-597146	− 21.6	2350 ± 30	2466–2329	94.7
					2486–2481	0.7
100	Bulk sediments	BETA-601091	− 20.3	2660 ± 30	2792–2739	77.1
					2848–2809	18.3
120	Bulk sediments	BETA-597147	− 19.1	3130 ± 30	3409–3319	64.7
					3304–3247	25.7
					3445–3423	5
140	Bulk sediments	BETA-601092	− 19.1	3730 ± 30	4154–3981	93.5
					4221–4208	1.9
160	Bulk sediments	BETA-597148	− 19.9	4080 ± 30	4650–4511	65.2
					4806–4756	14.6
					4485–4441	10.9
					4700–4671	4.7
200	Bulk sediments	BETA-597149	− 19.8	4220 ± 30	4760–4692	41.9
					4855–4797	39.9
					4679–4642	12.8
					4634–4626	0.8

### Paleoenvironmental proxy data

**Figure 4:**
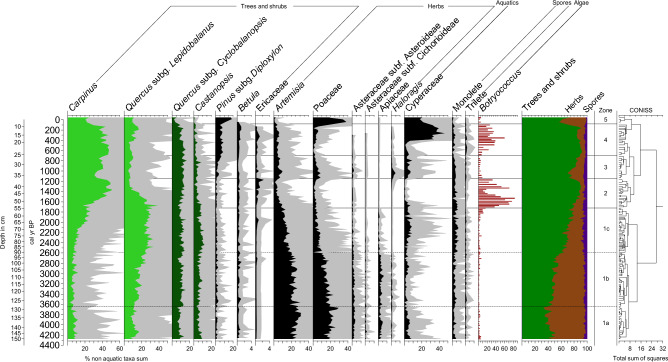
Selected pollen taxa from Dongsuak sediments. All percentages are calculated based on the total non-aquatic taxa sum. Deciduous and evergreen broadleaf taxa used for the TPIT calculation are colored in pale green and dark green, respectively. Gray shading indicates 5 × exaggeration. Note different units for *Betula* and Ericaceae.

**Figure 5:**
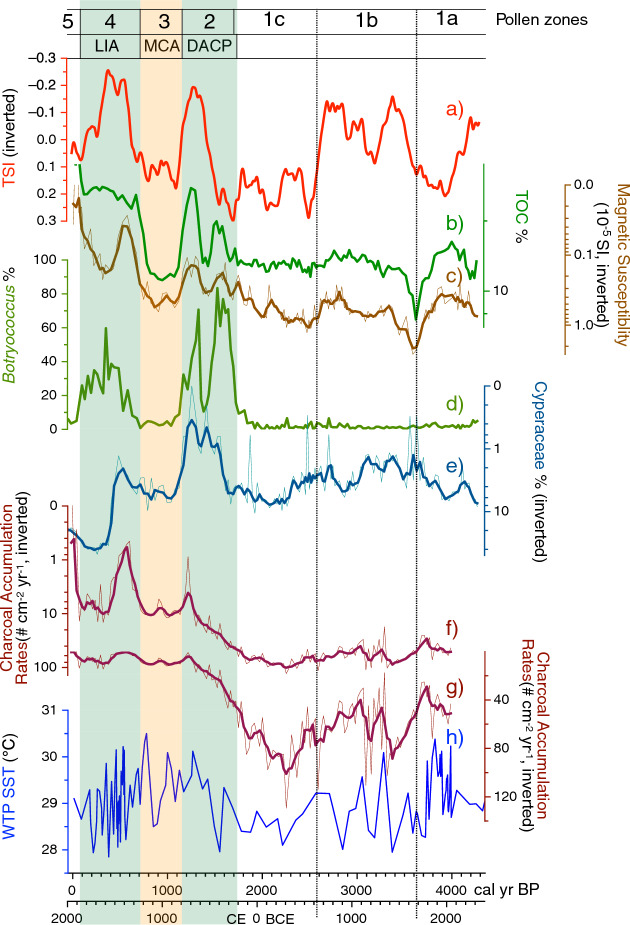
Comparison of the total solar irradiance (ΔTSI) data^23^ (250-year moving average) (**a**), Dongsuak multi-proxy data from this study (**b**–**g**), and reconstructed sea surface temperatures of the western tropical Pacific^24^ (**e**). Dry periods are highlighted by pale orange boxes, whereas wet periods are indicated by pale green boxes. Note that charcoal data were charted using a log scale on the y-axis (**f**), as well as a normal scale (**g**). *LIA* Little Ice Age, *MCA* Medieval Climate Anomaly, *DACP* Dark Ages Cold Period. This diagram was generated using pro Fit 7.0.19 software (www.quansoft.com).

**Figure 6:**
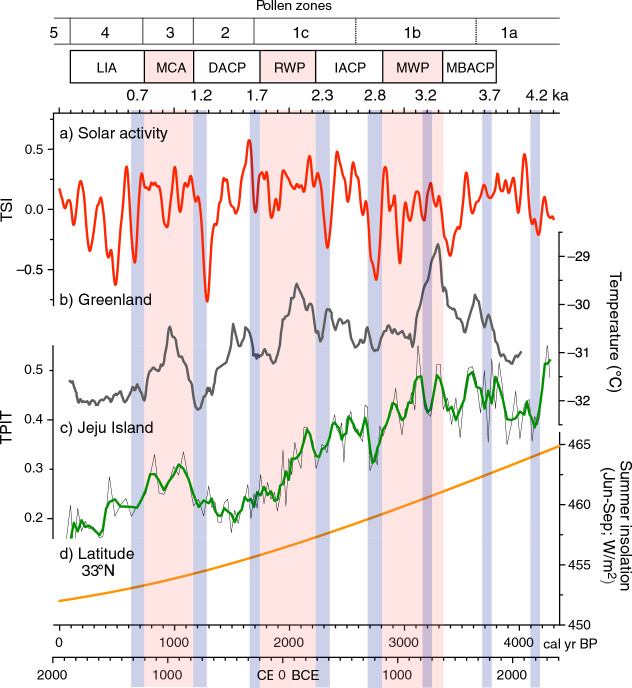
Comparison of the ΔTSI data^23^ (50-year moving average) (**a**), Holocene temperature reconstructions^26^ based on Greenland GISP2 ice core δ^18^O records (**b**), TPIT data (**c**), and Holocene summer insolation change at latitude 33°N (**d**). Cool periods are indicated by blue boxes, with their approximate ages; warm periods are indicated by red boxes. *LIA* Little Ice Age, *MCA* Medieval Climate Anomaly, *DACP* Dark Ages Cold Period, *RWP* Roman Warm Period, *IACP* Iron Age Cold Period, *MWP* Minoan Warm Period, *MBACP* Middle Bronze Age Cold Period. This diagram was generated using pro Fit 7.0.19 software (www.quansoft.com).

For ease of discussion, the pollen diagrams were divided into five zones and three subzones based on clustering results (Fig. 4). The same zones were also utilized to describe other proxy data (Figs. 5 and 6).

#### Pollen zone 1 (150–55 cm): 4350–1700 cal year BP

This zone was divided into three subzones. Zone 1a (150–128 cm) exhibited a high pollen percentage of herbaceous plants, including *Artemisia* and Poaceae, as well as aquatic plants such as Cyperaceae (Fig. 4). TOC percentages were relatively high, whereas MS values and CARs were low (Fig. 5). Notably, there was a negative correlation between TOC and MS throughout the sediment profile.

In zone 1b (128–90 cm), there were consistent decreases in *Artemisia* and Poaceae percentages, whereas *Quercus* subg. *Lepidobalanus* showed an increase. Cyperaceae frequencies were lower, and tree pollen index of temperature (TPIT) values were higher, compared with the previous subzone (Fig. 6). TOC percentages gradually increased, whereas MS values declined.

Zone 1c (90–55 cm) was characterized by a significant increase in *Lepidobalanus* percentages. Similar to zone 1b, *Artemisia* and Poaceae percentages steadily decreased. Cyperaceae frequencies and CARs both showed a substantial increase around 2300 cal year BP, followed by a gradual decline. TPIT and MS values consistently decreased.

#### Pollen zone 2 (55–37 cm): 1700–1150 cal year BP

In zone 2, the percentages of *Artemisia* and Poaceae dramatically decreased to ≤ 2%. *Lepidobalanus* and Cyperaceae also experienced substantial decreases. Conversely, *Carpinus* percentages substantially increased to nearly 50%, and *Botryococcus* greatly increased to 80%. TPIT values continued to decrease until 1500 cal year BP, then began to recover. CARs steadily decreased. TOC percentages were higher compared with the previous period, whereas MS values were lower. Notably, TOC, MS, and Botryococcus data all exhibited two peaks.

#### Pollen zone 3 (37–26 cm): 1150–700 cal year BP

All proxy records from zone 3 displayed trends opposite to the findings in zone 2. *Carpinus* abundance significantly decreased; herbaceous taxa (e.g., *Artemisia*, Poaceae, Asteraceae, and *Haloragis*) all increased. *Betula* and Ericaceae abundances declined compared with the previous zone. The percentage of Cyperaceae increased. However, *Botryococcus* abundance greatly decreased to 5%. TOC percentages were lower compared with the previous zone, whereas CARs, MS values, and TPIT values were all higher.

#### Pollen zone 4 (26–8 cm): 700–100 cal year BP

Zone 4 showed patterns similar to the findings in zone 2. The proportions of *Artemisia* and Poaceae decreased again. However, there were considerable increases in the abundances of tree taxa such as *Carpinus*, *Lepidobalanus*, *Betula*, Ericaceae, and *Pinus*. Notably, *Pinus* was rare in previous periods but began to show an increase in zone 4. Among the key tree taxa, only *Castanopsis* demonstrated a noticeable decline. *Botryococcus* displayed a sharp increase and reached 80%. Cyperaceae percentages and CARs, which remained low in the first half of the zone, rapidly increased beginning around 500 cal year BP. Conversely, both MS and TPIT values declined at the same time.

#### Pollen zone 5 (8 cm-surface): 100 cal year BP to present

In zone 5, human activities had a significant effect on vegetation. *Pinus* and herbaceous plants, especially Poaceae, displayed increasing importance. However, other tree taxa, Cyperaceae, and *Botryococcus* showed a considerable decrease. TOC percentages rose sharply, while MS values and CARs both declined.

### Paleoclimate proxy records of Dongsuak sediments

The Dongsuak proxy records, particularly the charcoal data, imply that mid-to-late Holocene hydroclimate variations in the study area were primarily driven by fluctuations in sea surface temperatures (SSTs) in the western tropical Pacific (WTP). The study area likely experienced drier periods when WTP SSTs were lower and wetter periods when WTP SSTs were higher.

The results of previous pollen studies in the Korean peninsula have suggested that the climate was comparatively dry during periods when WTP SSTs cooled because of long-term El Niño-Southern Oscillation-type variations^2,19–22^. The frequency of El Niño events may have increased, leading to reduced WTP SSTs and a smaller amount of atmospheric water vapor above the oceanic source region where the East Asian summer monsoon originates. Our CARs data support the notion that reduced precipitation, induced by lower WTP SSTs, resulted in more frequent and intense local wildfires in coastal East Asia, including the Korean peninsula and Jeju Island.

Comparisons between Dongsuak proxy records and 250-year moving averages of total solar irradiance (TSI) data^23^ suggest that varying levels of dryness or wetness in the study area were influenced by centennial-scale fluctuations in solar activity. Enhanced solar activity on this timescale resulted in drier conditions, and vice versa. These long-term shifts in TSI may have controlled multi-centennial, low-frequency variations in Holocene El Niño-Southern Oscillation; increased TSI likely triggered El Niño-like conditions and lower WTP SSTs^24^. The lower WTP SSTs may have led to decreased precipitation in the study area (Fig. 5).

The impacts of solar activity and WTP SSTs on the study area's hydroclimate are clearly captured in TOC, MS, *Botryococcus*, Cyperaceae, and charcoal records of Dongsuak sediments. TSI values were negatively correlated with TOC % and *Botryococcus* % over most of the period investigated, but positively correlated with Cyperaceae %, CARs, and MS values. During dry periods, likely triggered by low WTP SSTs, there would have been a decrease in both autochthonous productivity and the influx of allochthonous organic matter (indicated by low *Botryococcus* % and TOC %)^25^. Reduced precipitation could have led to more frequent wildfires (high CARs values), decreased tree density on the slope, and increased erosion (high MS values), in a successive manner. Additionally, increased influx of clastic materials into the lake may have caused a reduction in lake area, providing more space for Cyperaceae colonization.

The resemblance between our TPIT data and temperature reconstructions from Greenland ice cores^26^ suggests that TPIT can be used to gather local information regarding Holocene temperature change, although the data are not strictly quantitative. The TPIT records imply that temperature shifts in the study area were influenced by variation in both solar activity^27^ and summer insolation, which declined consistently during the mid-to-late Holocene (Fig. 6).

Our TPIT data, Greenland temperature reconstructions, and 50-year moving averages of TSI show significant similarities in periodicity, including ~ 1000-year warming cycles and ~ 500-year cooling cycles over the past 4000 years. These findings all indicate a connection between solar activity variability and mid-to-late Holocene temperature change in the study area (Fig. 6).

Intriguingly, the comparison between our proxy records and TSI data suggests that long-term (250 years) moving averages of TSI provide insights into hydroclimate change in the study area, whereas shorter-term (50 years) moving averages are more indicative of paleotemperature change.

### Hydroclimate changes since 2600 cal year BP

Our pollen data suggest that the study area became progressively wetter during the mid-to-late Holocene. A consistent increase in arboreal taxa and a decrease in non-arboreal taxa indicate forest expansion in and around the crater, supplanting grassland as rainfall increased. From ~ 2600 cal year BP to 1700 cal year BP (pollen zone 1c), trees such as deciduous oak and hornbeam became increasingly prominent in the study area, whereas the abundances of herbaceous plants decreased.

The causes of these observed changes (i.e., whether they were related to increased precipitation or higher temperatures) remain unclear. Considering the low WTP SSTs and high CARs in zone 1c, it is highly probable that the climate during this period was relatively dry with decreased precipitation (Fig. 5). Annual mean temperatures also appeared to decrease, consistent with a gradual decrease in summer insolation (Fig. 6). However, deciduous oaks exhibited a particularly competitive advantage over grasses during this period. Our findings suggest that deciduous trees benefited from a slight but steady increase in late Holocene winter insolation^19,28^ since 2600 cal year BP.

Additionally, high CARs in zone 1c indicate frequent wildfires because of the prevailing dry conditions between 2600 and 1700 cal year BP. These wildfires likely influenced slope stability, resulting in a significant influx of clastic materials into the lake. The increased area created by these deposits could have provided favorable conditions for colonization by sedges at the lake margin, as indicated by the increased prevalence of Cyperaceae.

From 1700 cal year BP to 1150 cal year BP (zone 2; DACP), precipitation increased, resulting in a wetter climate (as indicated by Dongsuak proxy records). Increased frequencies of *Botryococcus* and *Carpinus* suggest greater productivity in and around the lake. Furthermore, percentages of herb taxa, MS values, Cyperaceae percentages, and CARs all decreased during this period. These changes imply that the wet climate led to increased tree density, decreased wildfires, and reduced erosion. The wet conditions were likely related to a decrease in low-frequency solar activity and an increase in WTP SSTs.

There was a relatively warm period between 1150 and 700 cal year BP (zone 3), commonly referred to as the Medieval Climate Anomaly (MCA). However, hydroclimate conditions were particularly dry, as indicated by a reduction in the abundances of *Carpinus* and *Botryococcus*, a contrasting increase in frequencies of herb and aquatic taxa, and an increase in MS values and CARs.

During the subsequent period from 700 to 100 cal year BP (zone 4), corresponding to the Little Ice Age (LIA), wet conditions returned. Changes were evident in MS values, Cyperaceae percentages, and CARs. Notably, the first half of this period likely included a significant increase in precipitation, as implied by rapid declines in the values of all three proxies. However, the sudden rebound at ~ 1600 CE suggests a transition to drier conditions during the middle of the LIA, consistent with paleoenvironmental proxy records from Northeast China^29,30^ and the western tropical Pacific^31,32^.

**Figure 7:**
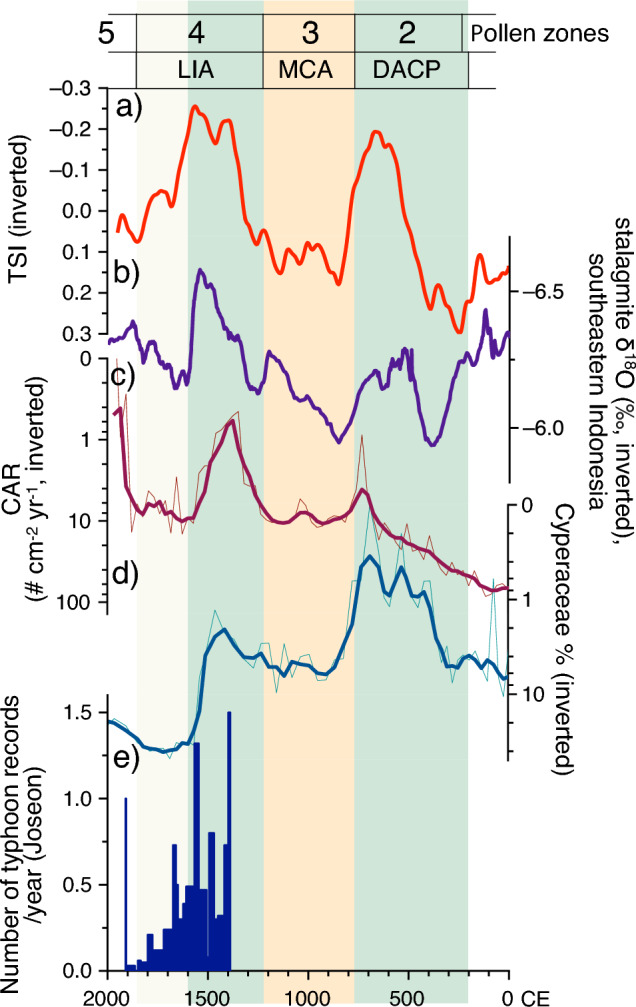
Comparison of the ΔTSI data^23^ (250-year moving average) (**a**), stalagmite δ^18^O records from Liang Luar, southeastern Indonesia^31^ (**b**), charcoal accumulation rates (CARs) from this study (**c**), Cyperaceae percentages from this study (**d**), and Joseon Dynasty records of typhoon landfalls^33^ (**e**). Dry periods are highlighted by pale orange boxes, whereas wet periods are indicated by pale green boxes. Beige box indicates drier conditions during the latter half of the LIA. *LIA* Little Ice Age, *MCA* Medieval Climate Anomaly, *DACP* Dark Ages Cold Period. This diagram was generated using pro Fit 7.0.19 software (www.quansoft.com).

For instance, western Pacific hydroclimate reconstructions from stalagmite δ^18^O data for southeastern Indonesia clearly demonstrated sudden drying at ~ 1600 CE (Fig. 7). The similarity between Indonesian data and Dongsuak records supports our conclusion that Holocene climate change on Jeju Island was primarily controlled by variations in WTP SSTs. Furthermore, the Annals of the Joseon Dynasty (official state records) indicate a dramatic decrease in typhoon landfalls beginning at ~ 1600 CE^33^. This information suggests that decreasing WTP SSTs suppressed typhoon generation, reducing the influx of water vapor into coastal East Asia (Fig. 7).

After ~ 1850 CE (zone 5), variations in the proxy data no longer indicate natural climate change but instead reflect the effects of human activities.

**Figure 8:**
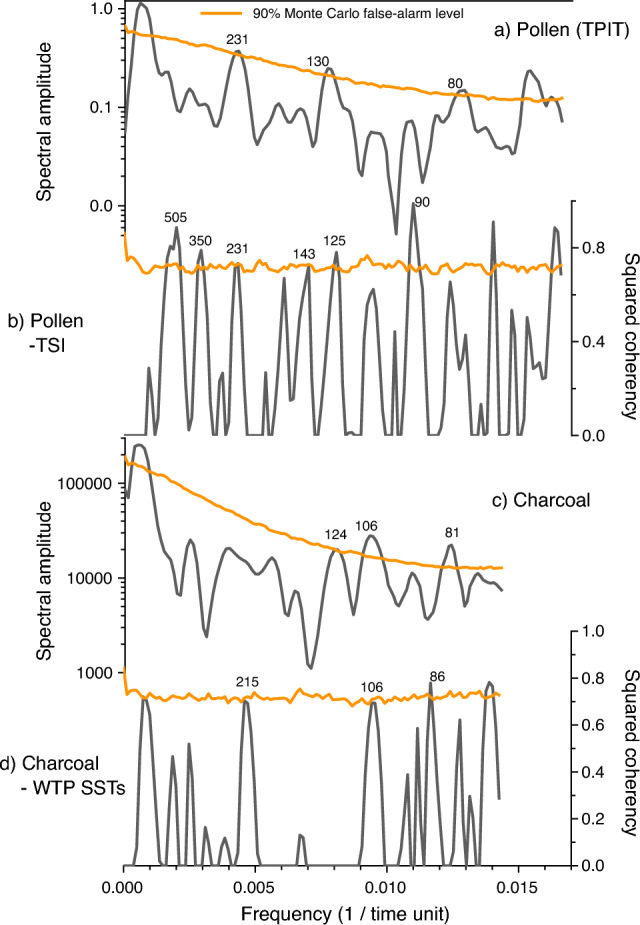
Autospectral analyses of TPIT data (**a**) and charcoal accumulation rates (CARs) data (**c**). Cross-spectral analyses between TPIT data and ΔTSI records^23^ (**b**) and between CARs data and SST reconstructions from the western tropical Pacific^24^ (**d**). Significant periodicities are labeled in years. This diagram was created using pro Fit 7.0.19 software (www.quansoft.com).

The results of autospectral analysis on charcoal data showed hydroclimate cycles of 124, 106, and 81 years, which were significant at the 90% Monte Carlo false alarm level. Additionally, there was significant statistical coherence between CARs and WTP SSTs for cycles of 215, 106, and 86 years (Fig. 8). These cycles closely resemble sunspot periodicity at 230-year^34^, 212-year^35^, 130-year^36^, 106-year^35^, and 88-year^35^ cycles. These findings indicate that hydroclimate variability in the study area, driven by variations in WTP SSTs, was connected with major cycles of solar activity.

### Past temperature variability

The Greenland temperature reconstructions^26^ and our TPIT data showed similar trends and fluctuations since ~ 4000 cal year BP. These changes were characterized by gradual cooling punctuated by periods of relative warmth occurring at ~ 1000-year intervals. The overall temperature decline was likely to have been connected to a consistent decrease in Holocene summer insolation. Both records also revealed short-term cooling periods at 400–600-year intervals, suggesting periodic climate variation in the study area. Paleoclimatological studies from various regions, globally, increasingly show abrupt climate changes, including late Holocene cooling events at 4.2 ka^37,38^, 3.7 ka^39^, 3.2 ka^40^, 2.8 ka^41,42^, 2.3 ka^43^, 1.8 ka^44^, 1.2 ka^45^, and 0.6 ka^46^. The ~ 500-year cyclicity is presumably associated with low-frequency sunspot variability^47^ and subsequent shifts in WTP SSTs^1,2,29^. Simultaneously, temperatures appear to have dropped at ~ 230-year intervals throughout the period investigated, potentially in relation to periodic changes in Holocene solar activity. The 210-year Suess/de Vries cycle has been detected most frequently in Holocene paleoenvironmental proxies^48^.

As previously mentioned, the study area appeared to experience warm periods at ~ 1000-year intervals. These are commonly referred to as the Minoan Warm Period (MWP, 3400–2800 cal year BP)^49^, Roman Warm Period (RWP, 2200–1750 cal year BP)^50^, and MCA (1200–800 cal year BP)^51^. Between these warm periods, there were relatively cool periods, such as the Iron Age Cold Period (IACP, 2800–2300 cal year BP)^52^, Dark Age Cold Period (DACP, 1750–1200 cal year BP)^53^, and LIA (700–100 cal year BP).

The autospectral analysis of our TPIT data revealed that the 230-, 130-, and 80-year periodicities were significant. These periodicities were similar to the 210-year de Vries cycle and 88-year Gleissberg cycle, both of which had substantial effects on global climate variability during the Holocene^54,55^. Additionally, key solar cycles were confirmed in the coherency spectrum between TPIT data and TSI records (Fig. 8). Coherent periodicities of 505, 350, 231, 125, and 90 years were significant at the 90% Monte Carlo false alarm level. They correspond to the solar cycles of 504, 355, 230, 130, and 88 years reported in previous studies^34–36,56^. These findings collectively suggest that changes in TSI were highly likely to be responsible for mid-to-late Holocene temperature shifts in the study area.

### Societal response to climate change

As previously mentioned, our TPIT data exhibited a ~ 500-year cycle of climate deterioration centered at 4.2, 3.7, 3.2, 2.8, 2.3, 1.8, 1.2, and 0.6 ka. Importantly, the cooling events at 2.8 ka and 2.3 ka are suspected to have significantly impacted ancient Korean societies. Recent Korean paleoenvironmental studies increasingly recognize these events, with time gaps of ~ 500 years between them^2,57^. Furthermore, Holocene WTP SST reconstructions^24^, TSI data^23^, and stalagmite δ^18^O records from Dongge Cave, China^58,59^, all revealed distinct changes around 2800 and 2300 cal year BP.

**Figure 9:**
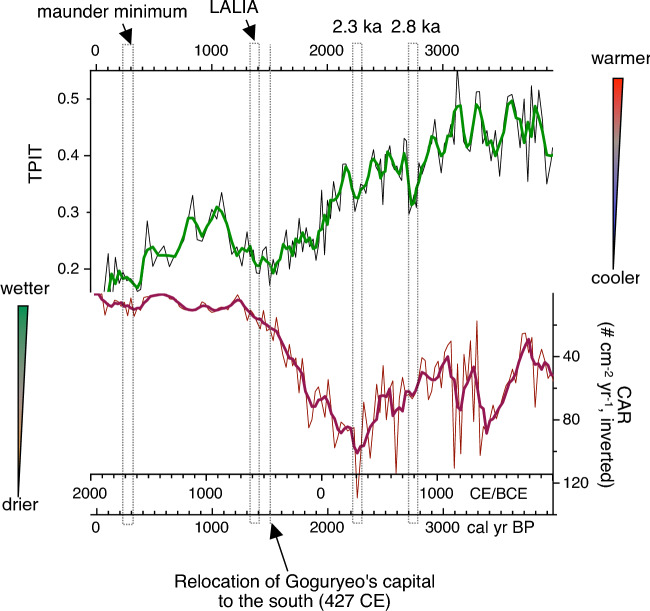
Late Holocene climate deteriorations (cooling and/or drying) and societal responses on the Korean peninsula. *LALIA* Late Antique Little Ice Age. This diagram was created using pro Fit 7.0.19 software (www.quansoft.com).

The amount of water vapor reaching the Korean Peninsula appears to have diminished during the 2.8 ka and 2.3 ka events, likely because of decreased WTP SSTs under sustained El Niño-like conditions. Specifically, around 2300 cal year BP, the study area experienced extreme dryness, as indicated by the highest CARs throughout the investigation period (Fig. 9).

Around 3000 cal year BP, a new group of farmers (known as the Songguk-ri assemblage) emerged in the southern Korean peninsula. The culture of these farmers, based on early rice agriculture, reached its peak 200 years later. However, the Songguk-ri societies, which once dominated the southern part of the peninsula, began to weaken around 2800 cal year BP and finally ended 2300 cal year BP. The abrupt disappearance of Songguk-ri culture is recognized as important by Korean archaeologists. Nevertheless, the exact cause of the culture's rapid decline remains unknown. Recent paleoclimate data from the Korean Peninsula suggest that the cause was climate deterioration around 2800 and 2300 cal year BP, likely to have been related to changes in WTP SSTs^2,22,57^.

During the warm periods, occurring at ~ 1000-year intervals, local societies may have remained stable because of reduced migration inflow. However, during the intervening cold periods, massive influx of migrating northern people might have caused chaos among the prehistoric societies of the peninsula. Asian paleoenvironmental studies increasingly suggest that late Holocene climate deterioration often drove northern people southward^60–64^.

During the Middle Bronze Age Cold Period (MBACP, 3800–3400 cal year BP), IACP (2800–2300 cal year BP), and DACP (1750–1200 cal year BP), people from Liaoxi and Liaodong likely migrated south to the peninsula. Conversely, during the MWP (3400–2800 cal year BP), RWP (2300–1900 cal year BP), and MCA (1200–750 cal year BP), local societies likely experienced stabilization because of the milder climate. Each cold spell presumably caused northern people to migrate southward, leading to internal and external conflicts in southern peninsula societies. This is not to say that climate change was the only factor driving these migrations and social upheavals. Many other factors were probably involved.

However, the northern people also brought advanced culture, which helped drive cultural development in the area. There is evidence that they introduced rice farming during the MBACP^65^, bronze dagger culture during the IACP^66^, and horse-riding culture during the DACP^67^. These material cultures helped the tribal societies of the Korean Peninsula to form ancient state systems.

In 427 CE, King Jangsu, who led the Kingdom of Goguryeo at its peak, made the decision to move the capital to Pyongyang. According to our TPIT data, the year of that move was within the coldest part of the DACP (Fig. 9). Therefore, it is difficult to dismiss climate change as a factor in King Jangsu's decision to move the capital over 200 km south of the original capital, away from the country's center. As the population grew, Goguryeo more heavily relied on crop farming; moving the capital to the warmer location of Pyongyang would have been a strategic decision in response to decreasing temperatures.

The DACP also includes the so-called Late Antique Little Ice Age (LALIA, 536–660 CE), identified through tree-ring data^68^. This period of climate deterioration, triggered by massive volcanic eruptions and lasting for over 120 years because of a subsequent decrease in solar activity, led to a series of social upheavals in Western Eurasia. In 541 CE, the Justinian Plague spread in the Eastern Roman Empire, causing tens of millions of deaths^69^. This pandemic may have been worsened by climate change and the resulting decline in crop yield. On the Korean Peninsula, the official historical record of the Three Kingdoms, the Samguk Sagi, shows that numerous people in the Kingdom of Goguryeo experienced severe famine in 536–537 CE. This tragedy was presumably caused by the volcanic eruptions of 536, which marked the onset of the LALIA^70^. Our TPIT data clearly record this drop in temperature during the mid-sixth century (Fig. 9).

The maunder minimum (1645–1715 CE), a period of extremely low sunspot activity, brought sudden famine to the Korean peninsula in 1670–1671 CE. The cold spring weather and typhoon-induced summer floods resulted in devastatingly poor crop yields. Additionally, because people displayed famine-induced deterioration of immunity, an epidemic of plague rapidly spread throughout the country, causing substantial population decline^71^. Korean historians refer to this disaster as the Kyungshin Great Famine. The low temperatures that likely led to lean years during the late seventeenth century are clearly visible in the TPIT data (Fig. 9).

In summary, cooling and drying events at 2.8 and 2.3 ka may have played important roles in the overall southward migration of Korean agriculturalists during the third millennium BP. The relocation of King Jangsu's capital at 427 CE, the Goguryeo famine at the beginning of LALIA, and the Kyungshin famine during the maunder minimum all seem to have been associated with a decline in temperatures.

## Conclusions

This study has focused on the reconstruction of mid-to-late Holocene climate changes in coastal East Asia using a high-resolution multi-proxy record from Jeju Island, Korea. Additionally, it explored the potential effects of abrupt short-term climate events on ancient societies in the Korean peninsula. This exploration was conducted by reconstructing hydroclimate and temperature variability over the past 4000 years. The primary study findings are summarized below.

First, our proxy records, particularly charcoal data, suggest that mid-to-late Holocene hydroclimate changes were primarily governed by SST variations in the WTP. A multi-centennial increase in TSI may have induced long-term El Niño-like conditions and lower WTP SSTs, which could have resulted in less precipitation in the study area. Overall, our records highlight a shift to drier conditions around 1600 CE during the middle of the LIA in the study area.

Second, temperature changes in the study area during the mid-to-late Holocene were largely driven by solar variability and a gradual decrease in summer insolation. Our TPIT data reveal a ~ 1000-year cycle of warming and a ~ 500-year cycle of cooling.

Finally, ancient societies on the Korean peninsula appear to have been significantly affected by abrupt short-term climate change events. Examples include the 2.8 ka event, the 2.3 ka event, the LALIA, and the maunder minimum. Notably, the decision to move the capital of Goguryeo, an ancient kingdom on the Korean peninsula, southward in 427 CE may have been influenced by a drop in temperature.

## Materials and methods

### Core materials and multi-proxy data

In June 2021, we retrieved a 300-cm-long sediment core from the Dongsuak swamp using a Russian-type peat corer (Fig. 2c). Nine bulk sediment samples and one macroscopic plant fragment were sent to Beta Analytic for accelerator mass spectrometry radiocarbon dating (Table 1). High δ^13^C values between − 20 and − 19‰ in the deeper samples suggest that C4 plants were dominant before C3 trees colonized inside the crater. We calculated the calibrated age ranges using rbacon 3.0.0 software^72^ and the IntCal20 dataset^73^. For this study, we utilized only the top 150 cm of sediments.

We collected 145 samples for pollen analysis at approximately 1-cm intervals from a depth of 3 cm to 150 cm. Pollen was extracted using standard palynological procedures^74^. The samples underwent sequential treatments with HCl, KOH, HF, and acetolysis; they were sieved with a 180-µm mesh filter to remove large debris after KOH treatment. Pollen counts were conducted on a Leica microscope with a 40 × objective at a total magnification of 400 ×. Each slide had a minimum of 300 pollen grains counted. We identified 95 pollen taxa, the alga *Botryococcus*, and two types of spores (monolete and trilete). We did not count other algae, such as *Pediastrum*, since their numbers were insufficient for any meaningful discussion. The frequency of *Botryococcus* is sometimes a very good climate-sensitive indicator of aquatic productivity^25^. It has been particularly useful in paleoenvironmental studies of sediment cores from Jeju Island^20,21,75,76^. A pollen diagram was produced using TILIA^77^. A stratigraphically constrained cluster analysis was done using CONISS. Pollen concentrations were calculated based on the ratios of added *Lycopodium* spores^78^. All percentages shown in the pollen diagram are based on the total sum of non-aquatic pollen and spores.

To reconstruct past local fire events, we counted > 125-μm macrocharcoal particles using a Leica EZ4 stereoscope^79,80^. This analysis was conducted for each depth between 6 and 140 cm, yielding 135 analyzed samples. For sample preparation, each sediment sample of 1.25 mL was soaked in H_2_O_2_ (6%) and incubated at room temperature overnight to bleach dark organic materials that could be mistaken for charcoal particles^80^. Then, materials < 125 μm and > 1 mm were discarded via sieving. Sieving with a grid size of 1 mm was conducted to remove only large plant fragments; it did not affect charcoal particles. The residual materials were poured into a Petri dish, and the number of particles bigger than 125 μm in axis length were counted manually for each sample without any weighting by further size fractions. Then, the counts were transferred to accumulation rates (# cm^−2^ year^−1^) using CharAnalysis ver. 1.1^81^.

MS was measured at 1-cm intervals using an MS2 meter (Bartington Instruments). TOC and total nitrogen were measured at the National Center for Inter-University Research Facility located at Seoul National University, Republic of Korea. Samples collected at 1-cm intervals from a depth of 3 cm to 150 cm were analyzed using the Flash 2000 CHNS/O Analyzer (Thermo Fisher Scientific, Bremen, Germany) with an accuracy of 0.3%. Prior to analysis, each sample was treated with HCl (10%) to remove inorganic carbonates.

### Tree pollen index of temperature

To estimate past temperature variability from the pollen record, TPIT values were derived using the following equation: (*Quercus* subg. *Cyclobalanopsis* + *Castanopsis*)/(*Quercus* subg. *Cyclobalanopsis* + *Castanopsis* + *Quercus* subg. *Lepidobalanus* + *Carpinus*). This index essentially calculates the ratio of evergreen broadleaf pollen to the total major tree pollen.

Previous studies, involving the collection and analysis of mosses from the slopes of Mt. Hallasan in Jeju Island^82^, revealed that *Castanopsis* pollen was predominant in moss samples from slopes below 450 m altitude, whereas pollen from *Quercus* subg. *Cyclobalanopsis* was prevalent in samples from 360 to 500 m altitude^82^. These broadleaved evergreens are characteristic of low elevation forests on Jeju Island. Conversely, deciduous tree taxa such as *Quercus* subg. *Lepidobalanus* and *Carpinus* were mainly observed in samples from higher-elevation areas between 600 and 1100 m^82^. The altitudinal distributions of these major tree species were consistent with the altitudes at which their pollen was deposited, confirming a strong correlation between the two parameters.

The arboreal pollen deposited over time on Dongsuak crater predominantly originated from four taxa: *Quercus* subg. *Cyclobalanopsis*, *Quercus* subg. *Lepidobalanus*, *Castanopsis*, and *Carpinus*. Because different types of trees flourish at specific altitudes based on their temperature preferences, the distribution of tree taxa on Mt. Hallasan follows an expected pattern. Altitude and temperature are inversely related; thus, we can qualitatively infer past temperature changes using TPIT data.

The proportion of broadleaf evergreen pollen may also be positively correlated with wetness. However, considering that Mt. Hallasan receives more precipitation at higher altitudes (Fig. 2a), we hypothesized that changes in the proportion of broadleaf evergreen pollen on the slope of Mt. Hallasan primarily reflect temperature variations. Additionally, we assumed that precipitation is negatively correlated with CARs because drought conditions tend to promote wildfires. Therefore, the analysis of Dongsuak sediments allowed us to reconstruct distinct mid-to-late Holocene shifts in temperature and precipitation.

### Autospectral analyses

We performed autospectral analysis on TPIT data using REDFIT software^83^. Three Welch-overlapped segment averaging segments (N50 = 3), 3 degrees of freedom (dofs = 3), 1000 Monte Carlo simulations (Nsim = 1000), and one Welch window (Iwin = 1) were used for all analyses. The TPIT records consisted of 145 data points with an average interval of 29 years between points [t(1) = 40 CE and t(145) = 4273 cal year BP]. We also conducted cross-spectral analyses using REDFIT-X software^84^ to assess climate cycles with significant coherency, comparing our TPIT data to TSI records, and our CARs data to WTP SST reconstructions.

## Data Availability

The datasets generated and/or analyzed during the current study are accessible upon reasonable request from the corresponding author.
